# Fibro-Adipogenic Progenitor Cell Alterations in Skeletal Muscle: Pathological Dysfunction or Adaptive Reprogramming?

**DOI:** 10.3390/ijms27115016

**Published:** 2026-06-02

**Authors:** Margarita Y. Sorokina, Oksana A. Ivanova, Anna A. Kostareva, Renata I. Dmitrieva

**Affiliations:** Institute of Molecular Biology and Genetics, Almazov National Medical Research Centre, 197341 Saint Petersburg, Russia; sorokinamy96@yandex.ru (M.Y.S.); oksana.ivanova.al@gmail.com (O.A.I.); akostareva@hotmail.com (A.A.K.)

**Keywords:** skeletal muscle, fibro/adipogenic progenitors, duchenne muscular dystrophy, skeletal muscle unloading, adipogenesis, myogenesis, metabolic flexibility

## Abstract

In skeletal muscle, there are two main progenitor populations crucial for growth, maintenance, and repair: satellite cells (SCs) and interstitial cells, of which fibro-adipogenic progenitor cells (FAPs) are the best characterized fraction. However, data on how specific diseases or physiological conditions affect the biological properties of FAPs are limited. In this review we analyze data obtained with FAPs purified from skeletal muscle tissue from Duchenne muscular dystrophy (both human patients and mdx mice models), hindlimb functional unloading (rats), and type 2 diabetes (T2DM, human patients). Here we discuss how disuse/disease affect FAP’s properties: the adaptive metabolic remodeling; the alterations in adipogenic differentiation in vitro; the possible role of particular subpopulations of FAPs in disease development; the role of FAPs in cell-to-cell interactions during skeletal muscle degeneration and regeneration. Current research has outlined how different physiological and pathological conditions alter FAPs’ behavior, highlighting FAPs as a potential target for clinical protocols aimed at treating or mitigating skeletal muscle disorders. Future studies should clarify how FAPs govern cell-to-cell interactions during skeletal muscle degeneration and regeneration, offering critical insights for therapies targeting diverse neuromuscular diseases.

## 1. Introduction

In healthy individuals, skeletal muscles exhibit significant metabolic flexibility in response to changes in physiological circumstances and external signals [1]. Being a major site of energy expenditure and metabolic activity, skeletal muscle can reshape its metabolism at the cellular level: different cell types may utilize distinct strategies of energy generation, primarily relying on glucose and/or fatty acids as key sources for ATP production [1].

The capacity to switch between fuels reflects skeletal muscle tissue metabolic fitness. At the cellular level, the carefully regulated glycolysis and oxidative phosphorylation fluxes are crucial for the processes that control the balance between proliferation and differentiation of skeletal muscle-resident stem cells during tissue development, growth, and regeneration. Therefore, changes in specific metabolic pathways, whether induced by disuse, aging, or neuromuscular and metabolic diseases alter the fate of both myogenic cells and interstitial cells: these disruptions impair proliferation and differentiation in the course of regeneration, ultimately leading to irreversible pathological changes in skeletal muscle, including fibro-fatty degeneration [2,3,4,5,6].

In skeletal muscle, there are two main populations of progenitor cells that are crucial for muscle tissue growth, maintenance, and repair: SCs [7] and interstitial cells, of which FAP cells are the best characterized population [8]. SCs play a key role in muscle post-injury regeneration, and they are the most studied skeletal muscle resident stem cells. Once activated, they proliferate, differentiate into myoblasts, and fuse with damaged muscle fibers to restore or replace them [9,10,11].

FAPs are the population of mesenchymal cells residing in the interstitial spaces within the muscle, which corresponds to the perimysium. Through the paracrine mechanisms and/or cell-to-cell contacts, FAPs dynamically regulate the microenvironment to support, depending on the physiological context, the preservation of SCs’ quiescence, activation of SCs’ proliferation, migration, and differentiation [12,13,14,15]. Upon injury, FAPs get activated to control the mechanisms involved in regulation of skeletal muscle regeneration and growth; thus, they undergo transient proliferation before being eliminated, thereby preventing pathogenic accumulation that could lead to fibrotic or fatty degeneration [16,17,18,19].

The experimental approaches to studying FAP’s functionality and metabolism are consistent across most of the works: muscle biopsy material is collected to purify and characterize the FAP population; subsequently, cells are expanded in vitro to obtain a sample size suitable for the specific experimental design. These projects typically utilize either animal models or patients’ muscle biopsies. The animal models of disuse-induced muscle atrophy are described in detail by Powers [20]; the information on animal models of inherited neuromuscular disorders is summarized by Vainzof and coauthors [21]. Notably, despite the numerous studies that utilize these models, there is very limited data on how particular diseases affect the specific biological properties of FAPs, and the specific stimuli that control coordinated SCs and FAP activation/inactivation are not yet completely defined. Also, it remains unknown if FAP-targeting metabolic changes are a cause or consequence of disease; it is accepted, though, that they are a key driver of fibro-fatty degeneration [15].

Importantly, there are recent works that confirmed with single-cell transcriptome sequencing (scRNA-seq) technology the heterogeneity of FAPs obtained from both healthy and diseased muscles, and showed disease-induced alterations in FAP subpopulations’ composition in DMD patients and T2DM patients; these data show the potential role of FAPs in the development of muscle degeneration and allow speculation that the particular subpopulations of FAPs might be responsible for certain functional disease-associated alterations in skeletal muscle tissue [22,23].

Thus, there is the need to better understand the role of specific skeletal muscle stromal cell subpopulations, in particular FAPs, in the pathogenesis of degenerative muscle diseases. In this work, we review the recently published data on the potential role of FAPs in the regulation of skeletal muscle regeneration and metabolic plasticity under altered physiological and pathological conditions. We also discuss which molecular mechanisms influencing FAP functionality could serve as targets for pharmacological treatments to counteract pathological changes in skeletal muscle tissue.

## 2. Alterations in FAPs Derived from Disuse- and Diseased Skeletal Muscle

Evidence suggests that FAPs can act as multifunctional sensors in skeletal muscle, translating regulatory signals into metabolic and functional reprogramming during both physiological and pathological conditions. Consequently, the changes in FAPs properties should result in skeletal muscle alterations.

### 2.1. FAPs in Muscle Atrophy Induced by Functional Unloading

Potentially reversible muscle atrophy is usually caused by factors that temporarily reduce muscle activity: limb immobilization (casts) after trauma, aging-associated sedentary lifestyle, periods of muscle disuse during recovery from illness or injury, or space flight leading to unloading of skeletal muscles. For these human conditions that result in muscle atrophy, a rodent model exists. For example, a tail suspension technique to unload the hindlimb muscles of rodents is frequently utilized to study the human muscle atrophy developing during space flight or bed rest/trauma-induced limb immobilization [20]. Importantly, even short-term bed rest rapidly induces muscle mass loss and decreases muscle oxidative capacity and whole-body insulin sensitivity. However, in contrast to obesity-induced insulin resistance, neither intra-muscular lipid accumulation nor a decline in the maximal activity of key mitochondrial enzymes was detected [24,25]. These observations indicate that the molecular mechanisms associated with muscle atrophy are cause-depended, and the specific molecular mechanisms supporting the reversibility of disuse-induced skeletal muscle alterations should be identified. To date, most studies in the field have relied on skeletal muscle biopsy samples to analyze disuse-induced changes in skeletal muscle tissue morphology, metabolism, contractility, and signaling pathways (see for review: [26]).

The works that investigate the disuse-induced alterations in FAPs most often are dedicated to atrophy associated with injury or skeletal muscle diseases, but not with temporary functional unloading [27]. Thus, the data on the function of FSPs in the course of skeletal muscle unloading are limited, and results are often controversial, most likely due to differences in experimental models and time courses of unloading.

**Figure 1:**
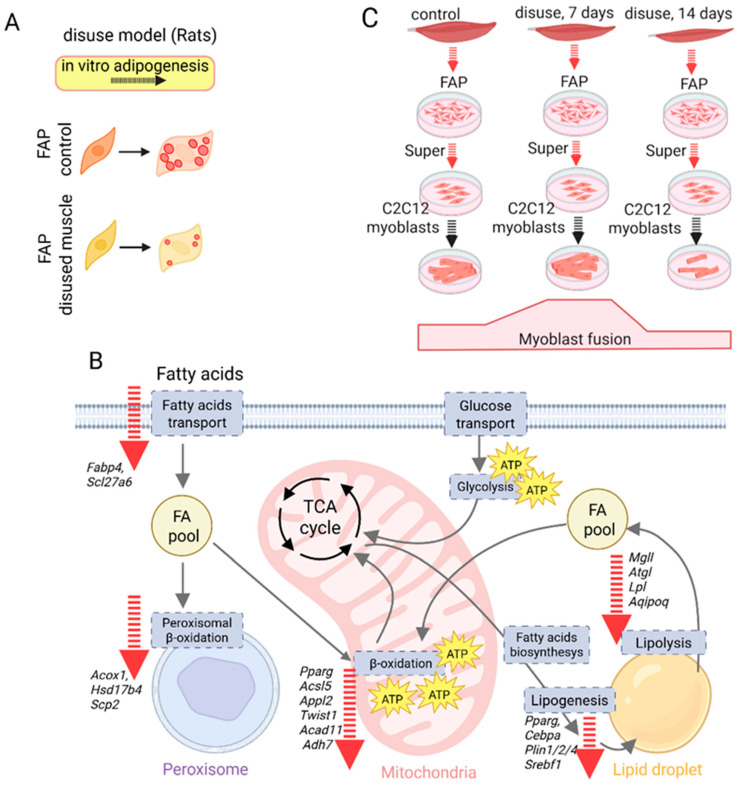
Metabolic and functional alterations in FAP associated with skeletal muscle disuse. (**A**) The adipogenic potential of FAPs purified from temporarily disused muscle gradually decreases over a 14 days unloading period; (**B**) The regulatory network that controls energy metabolism in temporarily unloaded rat m. soleus; red arrows indicate the downregulated pathways and specific gene expression; (**C**) Temporary skeletal muscle disuse affects FAPs’ ability to support differentiation of C2C12 mice myoblasts in the two phase-way: FAPs culture conditioned medium (super) collected from the control and 7-day disused muscle supports C2C12 in vitro myogenic differentiation, while super collected from FAPs purified from muscle disused for 14 days—does not support; (the schematic interpretation of data from [28] is presented; the figure is created in BioRender. Kostareva, A. (2025) https://BioRender.com/g535lfa).

In our work [28], performed with the hindlimb suspension model [20], our group analyzed the unloading-induced alterations in FAPs purified from healthy, temporarily inactive rat m. soleus muscle. Using a combination of transcriptome analysis and a number of functional in vitro assays, we detected the unloading-induced alterations in FAPs’ functioning after 7 and 14 days of unloading. For instance, FAPs purified from unloaded muscles exhibited suppressed adipogenic differentiation in vitro (Figure 1A); the analysis of the transcriptome confirmed that the unloading-induced metabolic alterations result in the decreased ability of FAPs to differentiate into adipocytes (summarized in Figure 1B). Specifically, in FAPs purified from functionally inactive muscle, the pathways that control lipid metabolic pathways were significantly downregulated: fatty acid (FA) transport, peroxisomal and mitochondrial beta-oxidation, lipolysis and lipogenesis, as well as the PPAR transcription factor signaling pathway that regulates the main steps of adipogenesis. In parallel, Seahorse analysis of live FAPs in real time revealed unloading-driven metabolic reprogramming. FAPs from inactive muscle exhibited decreased oxygen consumption rate (OCR), a measure of OXPHOS activity, while extracellular acidification rate (ECAR), a proxy for glycolysis, remained unchanged. Nevertheless, this adaptive decline in OXPHOS shifted FAPs toward predominant reliance on glycolysis compared to controls (Figure 1).

In a recent work [29] presenting the comprehensive skeletal muscle tissue atlas that integrates denervation-induced muscle atrophy and overloading-induced muscle growth within the same muscle type (plantaris), authors have shown the increased relative abundance of FAPs after 3 and 7 days of denervation compared to the basal condition, but the single cell/single nuclei transcriptome analysis (sc/sn RNA-Seq) have not revealed the substantial transcriptional changes in FAPs. In contrast, during muscle overloading FAPs showed the upregulated extracellular matrix (MMP14) and mechanosensory (Postn, Aspn, Adamtsl1, and Piezo2) genes, reflecting the potential for enhanced FAP-mediated support for myofiber growth [29]. Apparently, this discrepancy may reflect not only the differences between muscle types (soleus [28] vs. plantaris [29]), experimental approaches to transcriptome analysis (bulk RNA-Seq from in vitro-derived samples [28] vs. sn/scRNA-Seq for samples freshly purified from skeletal muscle [29]), but also the differences between rodent experimental models used. Consequently, hindlimb suspension model is more related to the bed rest after illness, aging-associated lifestyle, or space flight, all leading to unloading of skeletal muscle, while denervation model—to the neuromuscular diseases and progressive aging-related sarcopenia [30,31].

Perhaps aging is the best-described example of disuse-related progressive skeletal muscle atrophy. How aging alters FAPs’ activity has been reported in a few recent works [32,33,34]. Thus, in work performed with young (9–13 weeks old) and aged (20–25 months) mice [32], the immunostaining for FAPs marker (PDGFRα) revealed lower numbers of FAPs in the muscles of aged mice. Furthermore, as in the hindlimb unloading model [28], FAPs purified from aged skeletal muscle showed downregulated adipogenic differentiation in vitro under pro-adipogenic conditions compared to FAPs purified from young muscle [32]. Also, the authors showed that during muscle regeneration, FAP accumulation and clearance patterns differed between aged and young mice: aged mice had fewer FAPs at day 4 post-injury and, unlike young mice, failed to clear them by day 7 [32]. In contrast to FAP purified from muscle, unloaded for a short period [28], aged FAPs have displayed the reduced ability to support SC expansion and differentiation in vitro: the proliferation of SCs was increased in the presence of culture medium conditioned by young FAPs but was unaffected by medium conditioned by aged FAPs, which indicates that aging impairs the secretion of pro-myogenic supporting signaling molecules from FAPs. In this work, the authors propose a molecular mechanism for age-related skeletal muscle stem cells niche dysfunction. They show that FAP-secreted WISP1 (WNT1-inducible signaling pathway protein 1) declines with age. Restoring WISP1 (either by transplanting young FAPs or by systemic treatment) rescued muscle regeneration via Akt-mediated control of SC expansion and commitment [32]. Another mechanism of aging-related alterations in cells within supporting niches is highlighted in a number of works focused on the role of senescence in muscle regeneration [34,35,36,37]. Cellular senescence is a permanent, death-resistant arrest of dysfunctional cells. It occurs in development and injury, but can be harmful in aging tissues due to the release of inflammatory factors (senescence-associated secretory phenotype (SASP)) that impair regeneration. Senescent FAPs affect muscle homeostasis in different ways: they are cleared by macrophages during regeneration [37], but drive muscle degeneration in progeria that can be prevented by clearing senescent FAPs [38]. In a recent study published in Nature, Moiseeva and colleagues thoroughly examined senescent cells, including FAPs, in both young and old skeletal muscles [34]. Overall, in this work, they have shown that aging causes stressors that trigger inflammatory and fibrotic pathways, priming aged niche cells for senescence. Interestingly, the senescent cells were practically absent at basal conditions, even in old age, but emerged after injury [34]. The analysis of FAP transcriptome showed upregulated matrix remodeling, insulin signaling, and lipid metabolism genes, and downregulated mitochondrial genes in old versus young mice [34]. These aging-related alterations closely resemble the changes detected in FAPs during hindlimb unloading [28], except for the lipid metabolism pathways that were significantly downregulated during the unloading course. Senescent FAPs displayed altered lipid transport and contained more lipid droplets than non-senescent cells. Numerous lipid metabolism and lipid-transport genes, including Cd36 (a scavenger receptor linked to lipid metabolism and inflammatory function), were upregulated in all senescent cells [34,39]. They have also shown that transplantation of senescent cells (both FAP and SC) into the injured muscle of recipient mice delays regeneration. Moreover, the SASPs produced by senescent cells reduced SC proliferation in co-culture Transwell assays, but this effect was not observed when Cd36 was silenced (siCd36) in senescent cells before co-culturing with SCs; thus, CD36 was shown to be crucial for the paracrine effects of senescent cells on muscle regeneration by regulating SASP production.

In contrast, in experiments performed with a hind limb unloading model, FAP conditioned medium activated myogenesis in C2C12 myoblasts (determined by a wound healing assay, and myoblast fusion coefficient during in vitro myotube formation) if FAPs were purified from muscle and unloaded for a short period of time (7 days), but this stimulation declined subsequently by day 14 (see [28], and Figure 1C). Importantly, the upregulation of pro-fibrotic and pro-inflammatory pathways was not detected in unloaded FAP. This biphasic response to muscle disuse may reflect distinct stages of skeletal muscle adaptation to unloading, and the role of FAPs’ activity in the regulation of skeletal muscle adaptation: if the unloading persists, the adaptive response shifts toward preserving the skeletal muscle stem cell pool, especially the pool of SCs (See visualization on Figure 1C).

Collectively, the data discussed in this section indicate that muscle unloading caused by denervation, aging, or temporary disuse affects FAPs differently. In aged muscle, FAPs exhibit a reduced capacity to support muscle stem cells, whereas short-term functional unloading triggers a transient period of FAP activation that stops if unloading is prolonged. Denervation-induced disuse was accompanied by increased FAP abundance in muscle tissue, yet no transcriptional changes were detected, at least in the early stages. The analysis of these differences can be considered to develop the case-specific therapeutic and prevention strategies for skeletal muscle atrophy. For example, Pagano and co-authors demonstrated the beneficial effect of short-term muscle disuse following injury in a mouse model of muscle regeneration. They showed that a period of muscle rest after injury is necessary for more efficient muscle tissue restoration and reduced fatty infiltration, presumably via increased FAP apoptosis [40]. Or, for aging-induced atrophy, senolytic drugs can be considered a potential therapeutic/prevention strategy [41].

### 2.2. FAPs in Duchenne Muscular Dystrophy (DMD)

The most common and most studied type of progressive muscular dystrophy is Duchenne Muscular Dystrophy (DMD), caused by mutations in the dystrophin gene. The skeletal muscle fibers of both DMD patients and mouse DMD (mdx mouse model) show a reduced oxygen consumption and ATP production rate, defective oxidative phosphorylation, and skeletal muscle fatty and fibrotic degeneration [42,43]. Current data on the role of FAPs in DMD development and progression are limited; however, emerging evidence obtained with FAPs purified from both DMD patients and mdx mice support their contribution to DMD progression.

Importantly, the data obtained with both the mdx mouse model and human patients confirm the presence of distinct dynamic subpopulations of FAPs in skeletal muscle tissue. Thus, using the mdx mouse model, Malecova and co-authors described the skeletal muscle pro-fibrotic Vcam1+ FAP population, and the Tie2-high FAP population, associated with neonatal myogenesis [44]. Importantly, in this work, authors proved that the defective skeletal muscle regeneration and formation of fibrotic scars in DMD were associated with aberrant accumulation of Vcam1+ FAPs in injured tissue. Also, they have detected during the early stages of DMD progression the contrasting shifts in FAP subpopulations in the diaphragm muscle: in mdx mice pro-fibrotic Vcam1+ FAPs were enriched, while the Tie2-high FAP subpopulation was significantly reduced compared to wild-type controls [44]. This finding is important because the diaphragm, an involuntary muscle exposed to continuous contraction cycles critical for continuous breathing, is the earliest muscle to develop fibrosis during DMD progression [45].

In human, the single-cell RNA sequencing of FAPs derived from healthy donors and DMD patients also revealed distinct cellular subpopulations with specialized functions, such as muscle development regulation or cytokine activity control [23]. While universal markers like PDGFRA and COL1A1 were expressed across all clusters of FAPs, specific genes were upregulated in specific ones (IGFBP5 and CEMIP in myofibroblasts, HLA-DRA in regulatory, and CENPF in proliferative FAPs). The authors concluded, however, that these clusters represent dynamic, transitioning states rather than terminally differentiated cell types, as no highly specific transcriptional signatures were identified for each group. Importantly, while authors have shown the significant differences in the gene expression profile of DMD FAPs compared to control FAPs, this study does not provide evidence for differences in FAP cluster composition between DMD patients and healthy donors [23]. Thus, further research is needed to clarify whether alterations in specific FAP subpopulations are involved in DMD progression.

**Figure 2:**
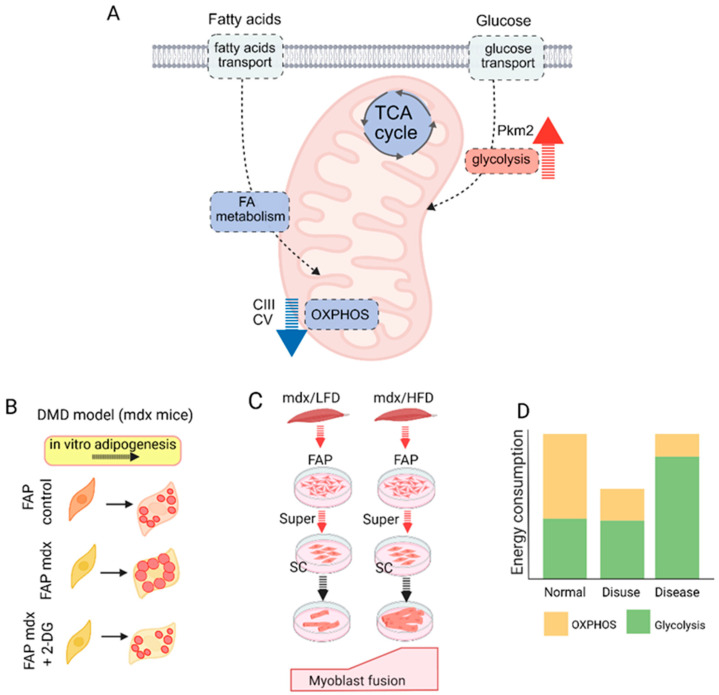
Changes in both energetic demand and nutrient supply affect mdx FAPs’ adipogenic potential and activity to support skeletal muscle regeneration through paracrine mechanisms. (**A**) The metabolic pathways are dysregulated in mdx FAPs: in red indicated the upregulated pathways/gene expression; in blue—downregulated; (**B**) Adipogenic differentiation in vitro is upregulated in mdx mouse—derived FAPs, and can be restored pharmacologically to the control levels; 2DG—2-Deoxy-d-glucose; (**C**) Sort-term high-fat diet (HFD) affected FAP functionality in mdx mice: skeletal muscle cultured satellite cells (SC) treated with cell culture medium (super) conditioned by FAPs from mdx mice fed with HFD showed a higher ability to differentiate, determined by fusion coefficient, than SC treated with super from FAPs derived from mdx mice on low-fat diet (LFD); (**D**) the balance between fractions of energy produced by glycolysis and OXPHOS in FAPs derived from mdx mice and unloaded muscle; (the schematic interpretation of data from [3,28] is presented; the figure is created in Biorender Kostareva, A. (2025) https://BioRender.com/nbny6bd (**B**,**C**) and BioGDP.com (**A**)). (FA—fatty acids; SC—satellite cells; 2-DG 2-Deoxy-d-glucose; LFD—low-fat diet; HFD—high-fat diet; Super—cell culture medium conditioned by FAP).

It is well established that DMD is associated with extensive metabolic disruptions in skeletal muscle tissue. Also, there is data indicating that FAPs derived from control and dystrophic (mdx) mice exhibit different patterns of metabolic profiles in vitro. Reggio and co-authors [3] reported the significant downregulation of enzymes involved in the regulation of FA oxidation, the Tricarboxylic Acid (TCA) cycle, and oxidative phosphorylation (OXPHOS), while glycolytic and pentose phosphate pathway enzymes were upregulated in FAPs purified from mdx mice. Specifically, both gene expression analysis and mass-spectrometry proteomic analysis showed that the key glycolytic enzyme pyruvate kinase M2 (PKM2) was significantly upregulated in dystrophic FAPs while mitochondrial complex V and III subunits were downregulated. This metabolic shift reflects a Warburg-effect-like glycolytic reprogramming (Figure 2A), which was associated with higher proliferation rates and increased adipogenic differentiation in mdx-derived FAPs compared to wild-type FAPs (Figure 2B), in contrast to FAPs purified from temporarily unloaded muscle, which exhibited unchanged glycolysis signaling pathways and downregulated adipogenesis (Figure 1A,B). Notably, modulating FAP metabolism by inhibiting glycolysis with 2-Deoxy-d-glucose (2-DG), reduced proliferation and restored normal adipogenic differentiation in dystrophic FAPs, as schematically summarized in Figure 2B. Importantly, the changes in the nutrients supply altered FAPs’ metabolism and affected FAPs’ ability to support skeletal muscle regeneration through paracrine mechanisms, which was shown for mdx mice [3]. First, FAPs purified from mdx mice fed a high-fat diet (HFD) had a higher basal oxygen consumption rate and ATP production than FAPs derived from mdx mice on a low-fat diet (LFD). Furthermore, skeletal muscle-cultured satellite cells treated with cell culture medium (super) conditioned by FAPs purified from HFD mdx mice demonstrated a higher ability to differentiate, determined by fusion coefficient, than SC treated with super from FAPs derived from LFD mdx mice (Figure 2C). Overall, HFD mdx mice showed decreases in the formation of fibrotic scars in the tibialis anterior (TA) and diaphragm muscles, while the limb injected with FAPs from HFD mice displayed an increased cross-sectional area of TA in comparison with the limb injected with FAPs from LFD mice. All these data support the assumption that metabolically reprogrammed FAPs can improve the dystrophic phenotype in DMD.

Thus, Reggio and co-authors [3] showed that HFD beneficially reprograms FAPs in skeletal muscle in Duchenne muscular dystrophy (Figure 2C). They have also suggested molecular mechanisms involved in this reprogramming: first, HFD provides “fuel” for mitochondrial pathways of fatty acid oxidation and TCA cycle; second, HFD represses the β-catenin inhibitors CK1 and MEST, and GSK-3β inhibition activates β-catenin signaling, which in turn modulates follistatin (Fst) expression, and Fst together with IGF1 promotes satellite cell differentiation and myotube hypertrophy, ultimately ameliorating the dystrophic phenotype [3].

The graph presented in Figure 2D schematically summarizes how FAPs respond to the changes that the skeletal muscle niche sends in order to activate the appropriate adaptive response. In case of disuse, when energy demand is temporarily decreased, FAPs downregulate the molecular mechanisms that control lipolysis, lipogenesis, and OXPHOS in order to decrease energy production and accumulation in adipose tissue. In progressive inherited muscle disease (DMD), FAPs undergo a Warburg-like glycolytic reprogramming in response to pathological microenvironmental signals. This adaptation drives hyperproliferation and fat accumulation, key features of the fatty-fibrotic degeneration.

Clearly, metabolic adaptability in skeletal muscle relies on the combined adaptive response of distinct cell types (satellite cells, myofibroblasts, myocytes, and FAPs), which is achieved through intercellular communication. Therefore, the evidence supporting the contribution of FAPs to muscle metabolic adaptation and regeneration suggests that these cells could serve as a potential therapeutic target in pathological conditions characterized by metabolic alterations, chronic or acute muscle loss, and fatty-fibrotic degeneration.

### 2.3. FAPs in T2DM

In Type-2 diabetes mellitus (T2DM), a metabolic disorder driven by obesity and physical inactivity, impairment in musculoskeletal health always is a hallmark of disease progression. Thus, the MRI and IH image analysis were employed to show that T2DM was associated with a gradual skeletal muscle fibro-fatty remodeling accompanied by skeletal fiber atrophic changes [46,47,48,49]. The role of FAPs in skeletal muscle fatty degeneration in T2DM was proved in experiments performed with KKAy mice, a genetically obese and diabetic model used to study type 2 diabetes, insulin resistance, and obesity [50].

Although studies that use FAPs purified from muscle tissue of T2DM patients are limited, important data have emerged from a primary cell culture-based in vitro model of T2DM. Hence, Farup and co-authors [22] provided the comprehensive description of the possible contribution of a particular subpopulation of FAPs to the development of skeletal muscle degeneration in T2DM patients. In this work, authors purified FAPs and isolated and described two distinct subpopulations of FAPs, defined by expression of surface marker THY1 (CD90): FAP^CD90+^/FAP^CD90−^ [22]. The wide spectrum of experimental approaches was employed to estimate the functional characteristics of these two subpopulations. FAP^CD90+^ cells showed a multipotential progenitor phenotype and authors suggested that they may have a central role in the development of the fibro-fatty phenotype. Based on results of clonal and limiting dilution assays, authors suggested that FAPCD^90−^ are committed toward adipocyte differentiation or have other stromal support functions. The real-time bioenergetics analysis showed the increase in both maximal oxygen consumption (OCR) and the reliance on glycolysis (increase ECAR) in FAP^CD90+^ cells compared to FAP^CD90−^ cells, suggesting a higher capacity for energy generation in FAP^CD90+^, high proliferative activity, clonogenicity, and production of extracellular matrix. Treatment with FAP^CD90+^ and metformin, an antidiabetic drug that increases the tissue sensitivity to insulin [48], significantly reduced oxygen consumption, but not glycolysis, reduced FAP^CD90+^ proliferation rate, and blocked the ability to form adipocytes in vitro. Importantly, metformin treatment reduced FAP content in the skeletal muscle of T2DM patients.

## 3. Could the Therapeutic Target Be Found Within Mechanisms That Control the Skeletal Muscle Interstitial Cells’ Functionality and Metabolism?

The functional and metabolic alterations detected in interstitial skeletal muscle resident cells in the response to the changes in physical activity, as well as the alterations induced by skeletal muscle inherited and metabolic diseases, may provide important and useful information regarding potential targets for therapeutic interventions aimed at controlling skeletal muscle fiber degenerative changes.

### 3.1. Metformin

Both dietary and pharmacological metabolic interventions can restore the bioenergetic balance, rewiring FAP phenotypical defects. As it mentioned above, Farup, J. et al. showed that the antidiabetic drug metformin markedly reduced oxygen consumption in cultured FAPs, blocked the increased ability of FAPs to proliferate and differentiate into adipocytes in vitro, and reduced FAP content in T2DM patients [22]. Based on those findings, authors suggested that the subpopulation of FAPs specifically marked by expression of THY1 (CD90+) could be the target for the treatment of impaired skeletal muscle function and fatty-fibrotic degeneration associated with T2DM [22].

**Figure 3:**
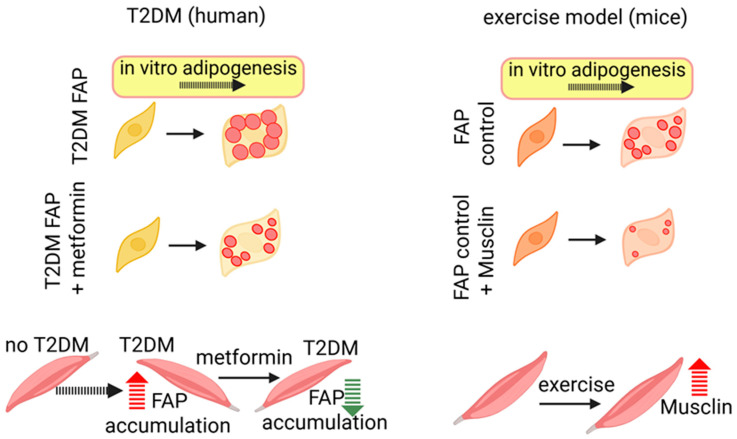
The therapeutic approaches to prevent FAP accumulation in skeletal muscle tissue. (**left**) Both FAP accumulation in skeletal muscle and in vitro pro-adipogenic activity decreased in metformin-treated T2DM patients; (**right**) exercise-stimulated myokine Musclin downregulates FAP adipogenesis in vitro; (schematically summarized data from [22,52]; (the figure is created in Biorender. Kostareva, A. (2025) https://BioRender.com/rdf28b0).

Based on those findings, authors suggested that the subpopulation of FAPs specifically marked by expression of THY1 (CD90+) could be the target for the treatment of impaired skeletal muscle function and fatty-fibrotic degeneration associated with T2DM [22]. To test if metformin indeed decreased FAP proliferation in vivo, authors obtained biopsy material from both T2DM patients and matched controls after 3 months of metformin (1000 mg metformin twice daily for three months) or placebo treatment, and estimated the FAP content in muscle using PDGFa expression as a marker of FAPs. Importantly, the increased FAP content was detected among the newly diagnosed, not treated, T2DM patients compared to healthy controls, while after 3 months of metformin treatment, the content of FAP was significantly reduced in T2DM patients. These data support the idea that metformin-induced activation of the AMPK-dependent fatty acid oxidation pathway provides mitochondria with new metabolic substrates to sustain the TCA cycle and ATP production [51], which may reduce the pathological FAP accumulation and skeletal muscle tissue remodeling, including fatty-fibrotic degeneration in T2DM patients. Therefore, the metformin treatment can be considered the strategy to reduce T2DM—induced skeletal muscle fatty-fibrotic degeneration (see Figure 3 for visualization).

While therapeutic strategies targeting metabolism (such as insulin sensitizers, PPAR agonists, Acetyl-CoA Carboxylase inhibitors, and AMPK activators) are well-established for common metabolic diseases like obesity and diabetes [53,54], emerging research suggests these targets also hold significant promise for treating neuromuscular disorders.

For instance, in 2021 Dong et al. showed that in the mdx mouse model of Duchenne muscular dystrophy (DMD), metformin improved muscle sarcolemma integrity, strength, and neuro-muscular junction (NMJ) transmission, providing new insights into metformin’s potential as a metabolic enhancer for DMD treatment [55]. On the other hand, the data obtained in clinical trials showed promising results, though not significant, improvements in DMD patients treated with metformin: (i) the combination of metformin with the nitric oxide precursor citrulline tested in a randomized, placebo-controlled phase 3 trial (47 patients; ClinicalTrials.gov NCT01995032, Updated 19 April 2018, Accessed November 2025) provided a clinically relevant but not statistically significant reduction in motor function decline [56]; (ii) the treatment of DMD patients with a combination of metformin/arginine (trial phase 1, proof-of-concept; 5 patients; ClinicalTrials.gov NCT02516085, Updated 7 July 2015, Accessed November 2025) showed delayed disease progression [57]. Further adequately powered research exploring this treatment option in DMD was proposed.

The discrepancies between existing lines of evidence indicate that further investigations are needed to find out how metformin treatment may affect the specific populations of skeletal muscle resident cells in progressive inherited skeletal muscle dystrophy. By now, it was shown that metformin treatment modulates the balance between SCs’ quiescence and activation [58,59], downregulates FAPs’ adipogenesis and proliferation in vitro, and prevents FAPs’ accumulation in skeletal muscle of T2DM patients [8,22].

### 3.2. Exercise and Muscline

The fact that exercise is a powerful tool for preventing and even treating metabolic diseases has been fully recognized only recently. Importantly, physical activity is the most adaptable component of energy expenditure: while the muscle contractions stimulate glucose disposal through insulin-dependent and independent mechanisms, the endurance training activates fat oxidation by increasing fatty acid transport and mitochondrial respiratory capacity [6]. The direct molecular link between exercise and FAPs activity was reported [52]. In that work, authors have shown that exercise-induced myokine Musclin controls the fate of FAPs and extracellular matrix remodeling via FILIP1L: both FAP accumulation in skeletal muscle tissue and FAP adipogenic capacity were attenuated by physical training; the exercise reduces the number of FAPs by upregulating FILIP1L, facilitates the clearance of apoptotic FAPs, and promotes muscle regeneration. Moreover, in vitro experiments showed the reduced proliferative activity of FAPs (confirmed by (BrdU labeling and expression of cell cycle biomarkers ccnd1, Cdca3, Cdc7Cdk1, Cdc20), as well as the ability to differentiate into adipocytes in vitro after Musclin treatment (confirmed by immunostaining for Perilipin and mRNA expression for Fabp4, Pparg, Plin1). Finally, authors performed the transcriptome and proteome analyses to confirm Musclin-FILIP1L connections with exercise-induced effects on FAP fate and muscle recovery and concluded that Musclin regulates the expression of CD47 through the FILIP1L/b-catenin pathway in apoptotic FAPs for macrophage clearance [47]. The Musclin-related decrease in the ability of FAPs to accumulate the lipids after the stimulation of adipogenesis [52] (Figure 3) is supported by the data obtained in experiments with cultured 3T3-L1 mouse adipocytes [60]: authors have shown that exogenous Musclin attenuates PKA-regulated p38 signaling, thereby ameliorating lipogenic lipid accumulation, but enhancing lipolysis in adipocytes.

Also, Musclin, an exercise-responsive myokine, amplifies natriuretic peptides (NPs) signaling. Unlike NPs, Musclin binds only Npr3 receptors, preventing the Npr3-mediated degradation of circulating ANP (atrial natriuretic peptide) and, therefore, increasing cyclic guanosine monophosphate (cGMP), which subsequently activates PGC-1α (peroxisome proliferator-activated receptor gamma coactivator 1-α) expression [61,62]. Thus, in an experimental mouse model, Musclin augments ANP signaling, leading to increased oxidative capacity and physical endurance in healthy skeletal muscle. In contrast, genetic disruption of Musclin production reduces exercise endurance, which is associated with a trend toward lower plasma ANP and significantly diminished post-exercise levels of skeletal muscle cGMP and PGC-1α [63]. In humans, Ji Sun Nam and co-authors [64] showed that aerobic exercises stimulate Musclin secretion, and the positive correlation was detected between Musclin and ANP levels during exercise and recovery, suggesting Musclin’s potential role in preventing ANP degradation.

Clinically, impaired NP-signaling in skeletal muscle is implicated in the pathophysiology of heart failure (HF), contributing to exercise intolerance and myopathy. Despite high systemic NP levels in HF patients, the beneficial effects on their skeletal muscle are blunted [62]. It was proposed that Musclin can locally counteract skeletal muscle dysfunction and exercise intolerance in HF patients. Indeed, the exogenous Musclin administered to heart failure mice with preserved ejection fraction (HFpEF) recovered impaired NP signaling and improved exercise capacity in the HFpEF experimental model [65]. Overall, the data on literature show the potentially dual function of Musclin in skeletal muscle physiology: the changes of its expression level may either reflect the adaptive response of the body to metabolic changes/demand or, as in T2DM, contribute to the disease progression by disrupting insulin signaling and inducing endoplasmic reticulum stress (ERS) (summarized in [66]). Additionally, musclin can act locally and have divergent effects depending on the type of cell. Even different subpopulations of FAPs could have specific, Musclin-induced responses. For instance, while in pro-adipogenic FAPs, exercise-induced musclin can activate the NP, system and induce lipolysis/inhibit lipogenesis to meet the increased need for energy in skeletal muscle tissue [60], while in pro-fibrotic FAPs, the musclin-induced decrease in proliferation activity and fibrotic remodeling can be detected [65].

To summarize this part: the future research should be done to understand Musclin’s role in the regulation of FAP-dependent skeletal muscle metabolism and physiology with a special focus on its local effects in skeletal muscle tissue, cell-type specific-effects, and its role in multi-organ communication.

## 4. Future Prospective

This review has outlined how different physiological and pathological conditions alter FAPs’ behavior, highlighting FAPs as a potential target for clinical protocols aimed at treating or mitigating skeletal muscle disorders.

In this context, future research focused on identifying the role of specific FAP subpopulations in the molecular mechanisms that regulate disease-induced FAP-mediated alterations in skeletal muscle tissue could help identify disease-specific therapeutic targets within distinct FAP subpopulations. To achieve this goal, reproducible 3D in vitro experimental models based on co-culture of myogenic progenitors with supporting cell types would be helpful [67]. This approach would add flexibility to experimental design, enabling a wide range of manipulations. For example, researchers could use genetically modified cells to model inherited skeletal muscle diseases [68,69,70], test damage from rare triggers such as snake venoms [71,72], and monitor the fate of each cell type during differentiation. These studies could clarify how FAPs govern cell-to-cell interactions during skeletal muscle degeneration and regeneration, offering critical insights for therapies targeting diverse neuromuscular diseases.

## Data Availability

No new data were created or analyzed in this study. Data sharing is not applicable to this article.
